# Identification and description of telerehabilitation assessments for individuals with neurological conditions: A scoping review

**DOI:** 10.1177/20552076231183233

**Published:** 2023-06-22

**Authors:** Jennifer O’Neil, Keely Barnes, Erin Morgan Donnelly, Lisa Sheehy, Heidi Sveistrup

**Affiliations:** 1 Faculty of Health Sciences, School of Rehabilitation Sciences, University of Ottawa, Ottawa, Ontario, Canada; 2 Bruyère Research Institute, Ottawa, Ontario, Canada; 3 The Ottawa Hospital Research Institute, Ottawa, Ontario, Canada; 4 Bayshore Home Care Solutions, Ottawa, Ontario, Canada

**Keywords:** telerehabilitation, remote assessment, outcome measures, neurology, rehabilitation

## Abstract

**Background:**

The clinical adoption of telerehabilitation accelerated rapidly over the last few years, creating opportunities for clinicians and researchers to explore the use of digital technologies and telerehabilitation in the assessment of deficits related to neurological conditions. The objectives of this scoping review were to identify outcome measures used to remotely assess the motor function and participation in people with neurological conditions and report, when available, the psychometric data of these remote outcome measures.

**Methods:**

MEDLINE (Ovid), CINAHL, PubMed, PsychINFO, EMBASE, and Cochrane databases were searched between December 13, 2020, and January 4, 2021, for studies investigating the use of remote assessments to evaluate motor function and participation in people with neurological conditions. An updated search was completed on May 9, 2022, using the same databases and search terms. Two reviewers independently screened each title and abstract, followed by full-text screening. Data extraction was completed using a pre-piloted data extraction sheet where outcome measures were reported as per the International Classification of Functioning, Disability and Health.

**Results:**

Fifty studies were included in this review. Eighteen studies targeted outcomes related to body structures and 32 targeted those related to activity limitation and participation restriction. Seventeen studies reported psychometric data; of these, most included reliability and validity data.

**Conclusion:**

Clinical assessments of motor function of people living with neurological conditions can be completed in a telerehabilitation or remote context using validated and reliable remote assessment measures.

## Introduction

Digital health is commonly used as an umbrella term describing the use of telecommunication technology within the healthcare context.^1^ In rehabilitation, digital health may involve the use of information and communication technology such as smartphone applications, wearable sensors, activity tracking devices, and telehealth platforms to assess, monitor, or deliver care. Telerehabilitation is defined as the use of communication technology to provide rehabilitation services including clinical assessments, health monitoring, and interventions.^2^ Although the adoption of telerehabilitation has increased dramatically over the last few years, rehabilitation professionals still report challenges associated with the uptake of telerehabilitation,^3,4^ documenting barriers such as remote assessment.

Outcome measures that are valid and reliable when used in remote assessments are necessary when documenting various motor deficits including balance, mobility, and upper extremity function. Currently, research has documented the psychometric properties of musculoskeletal assessments administered remotely (e.g., Oswestry Disability Index, goniometry)^5,6^; however, the lack of validated remote assessment measures for neurological deficits poses barriers to telerehabilitation implementation in neurological settings by limiting the ability of clinicians to objectively and accurately complete clinical assessment remotely.^7^ Authors have reported that remote monitoring and remote assessment are more difficult than in-person assessment due to perceived safety risks, limited space in home settings, or the necessity of caregiver presence.^8–10^ Although the evidence is growing rapidly for the validity of such remote assessments,^7,11,12^ additional research is necessary to identify new validated clinical tools.

The objectives of this scoping review were to: i) identify the remote outcome measures being used in the assessment of people with neurological conditions (PwN) including the description of the neurological conditions and the clinician characteristics, ii) describe, as per the International Classification of Functioning, Disability and Health (ICF), how these remote outcome measures were being used, and iii) report the psychometric data (e.g., validity, reliability, normative values) when available.

## Methods

### Systematic search

The complete scoping review methodology is reported in O’Neil et al.^13^ Briefly, following the methodology proposed by Arksey and O’Malley and adapted by Colquhoun et al.,^14,15^ an initial systematic search of MEDLINE (Ovid), CINAHL, PubMed, PSychINFO, EMBASE, and Cochrane (Appendix 1) was completed between December 13, 2020, and January 4, 2021. The search was updated on May 9, 2022. Specific search criteria were used to identify clinical outcome measures of motor deficits and function limitation of PwN (i.e., gait, balance, mobility, strength) conducted using telerehabilitation platforms including virtual reality, mHealth, videoconferencing, and digital health smart applications. Using Covidence online application (Melbourne, Australia), two reviewers (KB and EMD) independently screened article titles and abstracts to identify those meeting the inclusion criteria; two pairs of reviewers screened each article (KB, EMD, and JO). Full-text reviews were then completed by two reviewers (KB and JO) for articles meeting the review objectives. Conflicts were resolved by a fourth reviewer (LS) before moving to the next step. Interrater reliability between the three reviewers was assessed using Cohen's κ. The reference lists of all reviews and meta-analyses identified during screening were manually searched for additional articles. Since this scoping review did not involve human participants, participant consent was not necessary for this study.

### Data extraction

Two reviewers (JO and KB) extracted data from the included articles by categorizing the information into four main domains: i) general information (i.e., study design, sample size, clinician administering the assessment, and neurological condition); ii) outcome measures as per the ICF classifications of body function (i.e., physiological body functions) and activity and participation (i.e., task execution and life involvement); iii) teleplatforms used; and iv) psychometric data (i.e., validity, reliability, and normative data). The level of evidence for included studies was rated, when possible, using the method presented by Butler et al.^16^

## Results

### Included studies

**Figure 1.**
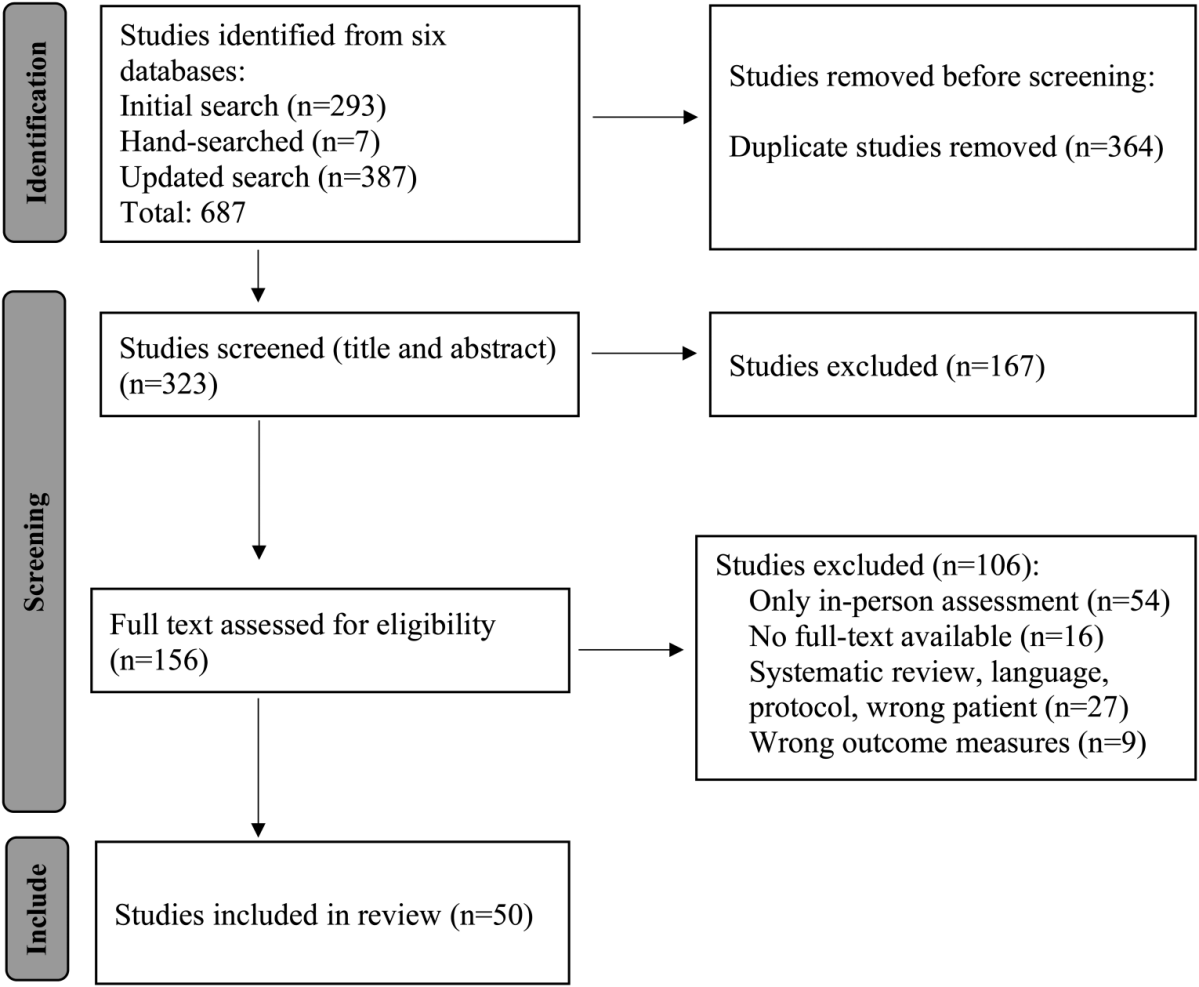
PRISMA flow chart reporting on study identification, screening, inclusion, and exclusion of studies (www.prisma-statment.org).

The systematic search identified a total of 687 articles. Two hundred and ninety-three articles were found during the initial search in 2020/2021 and a further 387 in 2022. After hand-searching the reference lists of reviews and meta-analyses identified during screening, an additional 7 articles were added. Of the 687 articles, 364 duplicates were removed, and 323 titles and abstracts were screened. Of the 323 articles screened, 167 were excluded, leaving 156 full articles to be reviewed. A further 106 articles were excluded once the full articles were reviewed, leaving 50 studies on the use of remote assessments for people living with various neurological conditions (Figure 1). Of these 50 studies, seven were among those added following the updated search, which suggested an increased interest in remote assessment since the onset of the COVID-19 pandemic. Interrater reliability for full article selection was very high (k = 0.86) between reviewers KB and EMD and high (k = 0.80) between JO and KB.^17^

### Objective 1: Identification of the neurological conditions assessed and the characteristics of the clinicians

The studies identified included those investigating remote assessment for people living with stroke (*n* = 17),^11,18–34^ Parkinson's disease (*n* = 16),^35–51^ traumatic brain injury including concussion (*n* = 8),^52–59^ multiple sclerosis (*n* = 6),^60–64^ and spinal cord injury (*n* = 3)^65–67^ (Table 1).

**Table 1.** Description of included studies.

References	Sample size	Design	Level of evidence	Population	Healthcare provider
Jack et al. 2001	*n* = 3	Pilot	V	Stroke	OT, PT
Zhang et al., 2001	*n* = 60 (30 with brain injury, 30 without)	Group comparison (patient to control)	II	Traumatic brain injury	Rehabilitation therapist
Theodoros et al. 2003	*n* = 10	Pilot	V	Acquired brain injury	SLP
Broeren et al., 2006	*n* = 0	Development of system	N/A	Stroke	Did not report
Fisher et al. 2007	*n* = 15	Pilot	V	Stroke	OT
Leocani et al., 2007	*n* = 24 (12 MS and 12 controls)	Group comparison (patient to control)	II	Multiple sclerosis	PT
Broeren et al., 2007	*n* = 5	Experimental, single-subject repeated design (AB)	IV	Stroke	OT
Giansanti et al. 2008	*n* = 3 (healthy)	Description of system and potential use	V	Parkinson's disease	Did not report
Kane et al. 2008	*n* = 20	Pilot	V	Multiple sclerosis	Physician's assistant with neurology experience and neurologist
Westin et al. 2010	*n* = 65	Description of system and potential use	V	Parkinson's disease	Nursing
Kowalczewski et al. 2011	*n* = 13	Experimental, RCT	I	Spinal cord injury	PT
Rábago et al. 2011	*n* = 1	Exploratory, case study	V	Traumatic brain injury (concussion)	PT
Wang et al. 2011	*n* = 54 (29 with PD and 25 controls)	Group comparison (patient to control), repeated measures	II	Parkinson's disease	Did not report
Arias et al. 2012	*n* = 34 (12 healthy young subjects, 12 elderly controls, 10 with PD)	Group comparison (patient to control), repeated measures	II	Parkinson's disease	Did not report
Combs et al. 2012	*n* = 9	Single-group cohort (pre-test/post-test)	IV	Stroke	Did not report
Cuthbert 2012	*n* = 20	Experimental, RCT (doctoral thesis)	I	Traumatic brain injury	PT
Westin et al. 2012	*n* = 30	Usability/Validation study	V	Parkinson's disease	Neurologist and neuropsychologist
Gilat et al., 2013	*n* = 28 (17 with freezing gait and 11 non-freezing gait)	Experimental, single-subject repeated measures design	IV	Parkinson's disease	Did not report
Thielbar et al., 2014	*n* = 16	Feasibility/Preliminary	V	Stroke	OT
Trincado-Alonso et al., 2014	*n* = 15	Feasibility	V	Spinal cord injury	Therapist
Arora et al., 2015	*n* = 20 (10 with PD and 10 controls)	Pilot	V	Parkinson's disease	Movement disorder specialist
Killane et al. 2015	*n* = 30 (13 with FOG and 7 without FOG)	Before and after intervention	III	Parkinson's disease	Movement disorder specialist
Sessoms et al. 2015	*n* = 25 (10 with mTBI and 15 health controls)	Group comparison (patient to control)	II	Traumatic brain injury (concussion)	PT program
Sola-Valls et al. 2015	*n* = 23	Longitudinal study over one year	V	Multiple sclerosis	Neurological examination
Sungmin et al. 2016	*n* = 9	Description and feasibility	V	Stroke	Did not report
Tobler-Ammann et al., 2016	*n* = 31	Validation	V	Stroke	OT
Trinh et al. 2016	*n* = 46	Dose-matched intervention study	IV	Stroke	Exercise physiologist
Wittmann 2016	*n* = 11	Single-group cohort	IV	Stroke	PT
Luque-Moreno et al., 2016	*n* = 2	Before and after intervention	III	Stroke	PT
Cubo et al., 2017	*n* = 40	Prospective randomized case–control	III	Parkinson's disease	Neurologist
Perez-Marcos et al., 2017	*n* = 10	Pilot	V	Stroke	PT
Vargas et al. 2017	*n* = 11 (male suspected concussion)	Feasibility	V	Traumatic brain injury (concussion)	Neurologist
Wright et al. 2017	*n* = 72 (14 with mTBI, 58 controls)	Group comparison (patient to control)	III	Traumatic brain injury (concussion)	Athletic trainer or PT
Finkelstein et al., 2018	*n* = 10	Feasibility	V	Multiple ssclerosis	PT and exercise physiologist
Salisbury et al. 2018	*n* = 42	Proof of concept study	N/A	Traumatic brain injury (concussion)	Did not report
Wall et al. 2018	*n* = 30	Feasibility	V	Multiple sclerosis	PT
Horin et al., 2019	*n* = 37 (17 intervention, 20 control)	Experimental, RCT	I	Parkinson's disease	OT, PT, SLP
Hung et al., 2019	*n* = 33	Experimental, RCT	I	Stroke	OT
Lheureux et al., 2020	*n* = 10	Pilot	V	Parkinson's disease	Did not report
Maillart et al. 2020	*n* = 46	Experimental, RCT	I	Multiple sclerosis	Neurologist
Ona et al. 2020	*n* = 20	Pilot	V	Parkinson's disease	Did not report
Thielbar et al., 2020	*n* = 20 (24 enrolled)	Experimental, RCT	I	Stroke	Research therapist
de Carvalho et al. 2021	*n* = 34	Observational/validation	V	Parkinson's disease	Trained researchers
Hawkins et al. 2021	*n* = 80 (40 with PD, 40 control)	Group comparison (patient to control)	III	Parkinson's disease	Did not report
Peters et al. 2021	*n* = 5	Pilot	V	Stroke	PT
de Rooij et al. 2021	*n* = 28	Experimental, RCT	I	Stroke	PT
Szturm et al. 2021	*n* = 10	Feasibility	V	Stroke	Did not report
An & Park, 2022	*n* = 40	Experimental, RCT	I	Spinal cord injury	PT
Borzì et al. 2022	*n* = 31	Description and use of technology	V	Parkinson's disease	Neurologist
Safarpour et al. 2022	*n* = 31	Feasibility	V	Parkinson's disease	Did not report

*Note:* OT: occupational therapist; PT: physiotherapist; SLP: speech language pathologist; RCT: randomized controlled trials.

Half of the studies (*n* = 25) included in this review were Level V evidence (i.e., case studies) and only eight were Level I evidence (i.e., RCT). Only 37 studies reported on the profession of the clinician completing the remote assessments; these included occupational therapists, nurses, movement disorder specialists, physiotherapists, speech-language pathologists, neurologists, athletic trainers, and exercise physiologists.

### Objective 2: Remote outcome measures described using the International Classification of Functioning, Disability and Health

#### Body structures

Eighteen studies investigated how remote outcome measures were implemented and used to assess deficits specific to body structure impairments. Investigations included the remote assessment of upper extremity motor impairments (*n* = 5), overall changes in range of motion, strength, or speed of muscle contraction (*n* = 5), condition-specific neurological impairment assessment (*n* = 4), lower extremity impairments (*n* = 2), and postural instability (*n* = 2). Teleplatforms included were virtual reality coupled with wearable sensors, asynchronous and synchronous telerehabilitation administered via videoconferencing, mobile applications, and home-motion sensors (Table 2A).

**Table 2A.** Remote assessments used to assess *body structure impairments* in various neurological conditions, following the International Classification of Functioning, Disability and Health.

References	Clinical assessment targeting body impairments	Telerehabilitation platforms
Stroke		
Jack et al. 2001	Range of motion Speed and strength of movement Finger fractionation	Virtual reality and wearable sensor: PC-based rehabilitation workstation (Pentium II 400 MHz PC with a FireGL 4000 graphics accelerator and two input–output gloves: CyberGlove and the Rutgers Master II-ND)
Fischer et al. 2007	Spasticity Isometric strength and velocity Range of motion	Virtual reality: Hand manipulator system, Virtual environment used CAVE Automatic Virtual Environment Library
Combs et al. 2012	Speed and smoothness of movement	Motion Monitor short-range transmitter system (Innovative Sports Training, Inc., Chicago, IL) with use of “mini-bird” sensors
Thielbar et al. 2014	Range of motion	Virtual Reality and wearable sensor: actuated virtual keypad (AVK) system with Cyberglove (PneuGlove) bend sensors (2000–0201, Flexpoint Sensor Systems, Inc.) located on the dorsal side of the glove
Peters et al. 2021	Motor assessment of lower extremity	Synchronous telerehabilitation: Fugl-Meyer telerehabilitation (FM-tele)
Parkinson's disease		
Westin et al. 2010	Motor assessment of upper extremity	Asynchronous: text messages
Wang et al. 2011	Motor assessment of upper extremity	Virtual reality: A projection-based VR system connected with the Patriot system was used. Open Graphics Library, Microsoft Visual and MFC
Arias et al. 2012	Motor assessment of upper extremity	Virtual reality: immersive experience a pair of Vuzix iWear VR920 glasses which includes motion tracking
Westin et al. 2012	Diagnostic and prognostic (i.e., assessment of symptoms)	Asynchronous: mobile Application
Cubo et al. 2017	Diagnostic and prognostic (i.e., assessment of symptoms)	Home-based motor monitoring: wireless motion sensor technology (KinesiaTM, Great Lakes)
Borzi et al. 2022	Postural instability	Wearable sensor: STMicroelectronics system-on-board prototype neMEMSi [27] was equipped with the following components: a 9-axis IMU (LSM9DS0)
Safarpour et al. 2022	Postural instability, Rigidity, Quality of gait	Wearable sensor: Opals by APDM Wearable Technologies, Portland, OR, USA
Multiple sclerosis		
Leocani et al. 2007	Motor assessment of upper extremity	Virtual reality and sensor: Visual feedback using dedicated software (Khymeia SRL, Padova, Italy) with magneto-electric sensor, (Polhemus Inc, Colchester, VT)
Kane et al. 2008	Diagnostic and prognostic (i.e., assessment of symptoms)	Synchronous videoconferencing
Finkelstein et al. 2018	Motor assessment of upper extremity	Synchronous: telerehabilitation (MS-Home Automated Telemanagement (HAT) system))
Maillart et al. 2020	Diagnostic and prognostic (i.e., assessment of symptoms)	Asynchronous: MSCopilot mobile application
Spinal cord injury		
Trincado-Alonso et al. 2014	Range of motion Diagnostic and prognostic (i.e., assessment of symptoms)	Virtual reality and wearable sensor: Virtual Reality (Toyra) and IMUs (MTx Xsens Company (Xsens Inc., Netherlands)
An et Park. 2022	Knee extension velocity	Virtual reality and wearable sensor

#### Activities and participation

Thirty-two studies reported on outcomes documenting changes in or status of activity limitations. No studies reported on outcomes specific to social participation restriction. Studies remotely assessed functional mobility, gait, and physical activity (*n* = 12), upper-extremity function including manipulation, grasp-and-release and reaching (*n* = 10), balance limitations (*n* = 7), and activities of daily living including communication activities and speech (*n* = 4). Virtual reality technology with or without wearable sensors or haptic systems was most frequently used, followed by wearable sensors combined or not with mobile applications and synchronous telerehabilitation (Table 2B).

**Table 2B.** Remote assessments used to assess *activity limitation* in various neurological conditions as per the International Classification of Functioning, Disability and Health.

References	Clinical assessment targeting activity limitation	Telerehabilitation platforms
Acquired brain injury (ABI)		
Theodoros et al. 2003	Speech and communication	Synchronous telerehabilitation (teleplatform)
Cuthbert 2012	Balance	Virtual reality: Wii Balance board
Traumatic brain injury (TBI) (including concussion)		
Zhang et al. 2001	Activity of daily living	Virtual reality: SOFTHAVEN (LinCom, Houston, TX)
Rábago et al. 2011	Balance	Virtual reality: CAREN System
Sessoms et al. 2015	Balance	Virtual reality: CAREN System
Vargas et al. 2017	Balance	Synchronous telemedicine: Telemedicine robot (VGo; VGo Communications, Inc., Nashua, NH)
Wright et al. 2017	Balance	Virtual reality: Visual stimulation (game)
Salisbury et al. 2018	Balance	Virtual reality and wearable sensors: COTS head-mounted wearable smart glasses
Stroke		
Broeren et al. 2006	UE function (i.e., manipulation, grasp, release, reaching)	Wearable sensors: Device recording all positions, movements, and forces, which are stored in the computer for further processing, analysis, and assessment.
Broeren et al. 2007	UE function (i.e., manipulation, grasp, release, reaching)	Virtual reality and haptic: Computer instrumentation haptic, force feedback and 3-dimensional visualization applied to computer games)
Sungmin et al. 2016	UE function (i.e., manipulation, grasp, release, reaching)	Virtual reality: Virtual BBT
Luque-Moreno et al. 2016	Functional mobility and gait	Virtual reality and wearable sensors: Wearable Sensors adapted Virtual Reality Rehabilitation System (VRRS), 3D motion-tracking system (Polhemus FASTRAK® 3Space, Vermont, USA)
Tobler-Ammann et al. 2016	UE function (i.e., manipulation, grasp, release, reaching)	Virtual reality and haptic: Handle instrumented with force sensors mounted on a PHANTOM Omni haptic device (Geomagic, USA)
Trinh et al. 2016	Functional and ambulatory movements- stepping, UE movements	Virtual reality: Wii Balance board
Wittmann et al. 2016	UE function (i.e., manipulation, grasp, release, reaching)	Virtual reality and wearable sensor: ArmeoSenso is a motion capture system based on wearable sensors in combination with an all-in-one touch screen computer (Inspiron 2330, Dell Inc., Fig. 1(a)).
Perez-Marcos et al. 2017	Functional mobility and gait	Virtual reality: Tabletop version of Mind-Motion™ PRO (MindMaze SA, Switzerland)
Hung et al. 2019	UE function (i.e., manipulation, grasp, release, reaching)	Virtual reality and wearable sensor: Accelerometer to ActiLife software, Kinect2Scratch Game
Thielbar et al. 2020	UE function (i.e., manipulation, grasp, release, reaching)	Virtual reality: Virtual Environment for Rehabilitative Gaming Exercises (VERGE)
de Rooij et al. 2021	Functional mobility and gait	Triaxial accelerometer (DynaPort MM, McRoberts BV, the Hague, the Netherlands)
Szturm et al. 2021	UE function (i.e., manipulation, grasp, release, reaching)	Virtual reality with sensor: Computer game-assisted tele-rehabilitation platform (GTP) with a novel application of a miniature inertial computer mouse (IB mouse)
Parkinson disease		
Giansanti et al. 2008	Physical activity (step count)	Wearable sensor: Sensor embedded in a band using a force sensing resistor
Gilat et al. 2013	Functional mobility and gait	Virtual reality: system not specified
Arora et al. 2015	Functional mobility (fine motor) and gait (reaction time, gait)	Mobile application: Software developed by the research team (MAL.)
Killane et al. 2015	Functional mobility and gait (dual tasking)	Virtual reality: Virtual corridor
Horin et al. 2019	Functional mobility and gait	Wearable sensor and mobile application: wGT3X-BT Activity Monitor, Actigraph, FL, USA with a three-axis accelerometer
Lheureux et al. 2020	Functional mobility and gait	Virtual reality: Homemade environment created with Unity software (USA) and written in C# (Visual Studio, Microsoft, United States; iVR headset (HTC, Vive, Taiwan), and Inertial Measurement Units. (IMeasureU Research, VICON, United Kingdom)
Ona et al. 2020	UE function (i.e., manipulation, grasp, release, reaching)	Virtual reality: Immersive virtual reality
de Carvalho Lana et al. 2021	Physical activity (step count)	mHealth devices (Google Fit, Health, STEPZ, Pacer, and Fitbit INC®)
Hawkins et al. 2021	Balance	Immersive virtual reality standaing balance test
Multiple sclerosis		
Sola-Valls et al. 2015	Physical activity (step count)	Wearable sensor and mobile application: Portable triaxial accelerometer (Actigraph GT3X)
Wall et al. 2018	Speech and communication	Mobile application: Cognitive Assessment for Aphasia App (C3A)
Spinal cord injury		
Kowalczewski et al. 2011	UE function (i.e., manipulation, grasp, release, reaching)	Virtual reality: ReJoyce (Rehabilitation Joystick for Computer Exercise) automated hand function test (RAHFT)

### Objective 3: Psychometric data

Out of 50 studies, 17 studies provided psychometric data for the remote outcome measures used. These included test–retest reliability of remote assessment, validity, and correlation between remote and in-person or traditional assessment as well as information around usability, acceptance, and satisfaction. A single study provided minimal clinical important difference (MCID) and normative values. Specifically, a cutoff score of steps per day was documented using wearable sensors that enabled the differentiation of ambulatory status in people living with Parkinson's disease^62^ (Table 3).

**Table 3.** Available psychometric data for remote assessments of neurological conditions.

References	In-person assessment	Remote assessment/Telerehabilitation platforms	Psychometric data
Theodoros et al. 2003	Frenchay Dysarthria Assessment (FDA)	FDA/ Synchronous videoconferencing	**Internal consistency reliability (ICC-95%CI):** 90% level of agreement between in-person and remote FDA assessment for the overall rating of severity of dysarthria.
Kane et al. 2008	Kurztke Expanded Disability Status Scale (EDSS)	EDSS/ Synchronous videoconferencing	**Interrater reliability (Cronbach's alpha):** Strong correlations between in-person and remote assessor (coefficients between 0.96 and 0.99) with respect to the EDSS total score.
Arias et al. 2012	NA	Finger tapping/ Immersive virtual reality (Vuzix iWear VR920 glasses)	**Test**–**retest reliability (ICC-95%CI):** Tapping Frequency for all groups (ICC 0.94) **Internal consistency reliability (ICC-95%CI):** Virtual reality between in-person assessors excellent for Tapping Frequency for all groups (ICC 0.98)
Cuthbert 2012	Berg Balance Scale (BBS)	Wii score/ Virtual reality: Wii Balance Board	**Validity (spearman correlation coefficient):** Between Wii Balance board tilt and BBS ranged from 0.28 and 0.68 (Static balance)
Westin et al. 2012	Unified Parkinson Disease Rating Scale (UPDRS), Hauser diary	Test battery/ Mobile application	**Internal consistency reliability (Cronbach's alpha):** Test battery (0.85) **Test–retest reliability (ICC-95%CI):** For the combined group 0.88 **Validity (Pearson's correlation coefficient):** Between Mobile App and UPDRS (*r* = −0.64) Between Mobile App and Hauser diary (*r* = 0.35)
Arora e al. 2015	UPDRS	Remote modified UPDRS/ synchronous videoconferencing	**Sensitivity:** The mean sensitivity in discriminating people with PD from controls using remote UPDRS was 96.2% (SD 2%) **Specificity:** The mean specificity in discriminating people with PD from controls using remote UPDRS was 96.9% (SD 1.9%)
Sola-Valls et al. 2015	6 Minute Walt Test (6MWT) EDSS	6MWT and EDSS /A portable triaxial accelerometer (Actigraph GT3X) and telephone	**Interrater reliability (**Kappa coefficient 95% CI)**:** Multimedia EDSS (total sample) vs in-person EDSS (0.4) Telephonic EDSS (total sample) vs in-person EDDSS (0.3) **Validity (Pearson's correlation coefficient):** Strong correlation between the MWT accelerometer and the in-person 6MWT (*r* = 0.76) **Cutoff score:** A cutoff of 3279.3 steps/day allowed to discriminate between patients with ambulatory impairment and fully ambulatory
Tobler-Ammann et al. 2016	Box and Blocks Test (BBT), Nine-hole Peg Test (NHPT)	Virtual Peg Insertion Test (VPIT)/ Virtual reality	**Test**–**retest reliability (ICC-95%CI):** Good to high overall test–retest reliability (ICCs ranging from 0.83 to 0.94). Fair reliability for grasping force (ICC = 0.75) and trajectory error (0.70) Poor reliability for trajectory error [cm] (0.67) and number of dropped pegs (0.58) **Concurrent validity (Pearson's correlation coefficient):** Low correlations between the VPIT and the NHPT (ρ = 0.31 for time; ρ = 0.21 for number of dropped pegs) Low correlations between the VPIT and the BBT (ρ = −0.23 for number of transported cubes; ρ = −0.12 for number of dropped cubes).
Sungmin et al. 2016	Box and Blocks Test (BBT)	Virtual BBT (VBBT)/ Virtual reality	**Validity (Pearson's correlation coefficient):** Strong correlations between the in-person BBT and virtual VBBT for nonhemiplegic (*r* = 0.904, *p* = 0.001) and hemiplegic sides (*r* = 0.788, *p* = 0.012)
Wittmann et al. 2016	Fugl-Meyer Assessment upper extremity (FMA-UE)	FMA-UE/ Virtual reality with wearable sensor (ArmeoSenso comprises a motion capture system based on wearable sensors)	**Validity (Pearson's correlation coefficient):** Significant correlation between the in-person FMA-UE and the automated FMA-UE with the number of workspace voxels (*r* = 0.91, *p* < 0.001), number of reached targets (*r* = 0.96, *p* < 0.001), and time to reach target (*r* = 0.92, *p* < 0.001)
Vargas et al. 2017	Standardised Assessment of Concussion (SAC), King-Devic test (K-D), Modified Balance Error Scoring System (mBESS)	SAC, mBESS, K-D/ Synchronous telemedicine robot (Vgo)	**Mean difference (seconds):** 100% agreement between in-person K-D and remote telemedicine K-D within 3 s differences (95% CI). 100% agreement between in-person SAC and remote telemedicine SAC (95% CI). 100% agreement between in-person sideline mBESS scores and remote mBESS score within 3 points (6/6; 95%CI 54%–100%).
Wright et al. 2017	K-D, Balance Error Scoring System (BESS), Oculomotor Tests	VR-Balance/ Virtual reality: Visual stimulation (game)	**Validity (Pearson's correlation coefficient)** The VR-balance test correlated most significantly with: the gaze stabilization test (*r*max = 0.55) the optokinetic stimulation (*r*max = 0.40) the horizontal eye saccades (*r*max = 0.41) No correlation with near-point convergence distance, King-Devick total time, dynamic visual acuity test, and total BESS score
Wall et al. 2018	Cognitive Assessment for Aphasia Paper-test	Cognitive Assessment for Aphasia App (C3A)/ Mobile application	**Feasibility and acceptability** Administration time for the C3A was approximately 20 min. No statistical difference (*p* = 0.38) in participant preferring C3A over paper-test between the aphasia (71%), stroke nonaphasia (76%) and control groups (59%), respectively.
Maillart et al. 2020	Multiple Sclerosis Functional Composite (MSFC)	MSFC APP/ Mobile application (MSCopilot)	**Test**–**retest reliability (ICC-95%CI)** Good reproducibility of MSCopilot (ICC = 0.90; *p* < 0.001) **Validity (Pearson's correlation coefficient)** Strong correlation between the digital and in-person MSFC test scores (*r* = 0.81; *p* < 0.001) **Satisfaction** The majority of patients preferred the use of the mobile application over traditional tests.
Ona et al. 2020	Box and Blocks Test (BBT) Hoehn and Yarh scale	BBT/ Immersive virtual reality	**Test**–**retest reliability (ICC-95% CI)** Excellent correlation between the two attempts for the most affected side (ICC = 0.876) and for the less affected side (ICC = 0.873) **Validity (Pearson's correlation coefficient)** Statistical significance and moderate correlation between BBT and VR-BBT for more affected side (*p* = 0.025, *r* = 0.499) and less affected side (*p* = 0.022, *r* = 0.510) Statistical significance and moderate correlation between Hoehn and Yahr scale and the VR-BBT for: more affected side (*p* = 0.001, *r* = 0.496) and less affected side (*p* = 0.038, *r* = 0.696) **Usability and acceptability** High to excellent perceived clinician usability and acceptability of the VR-BBT
de Carvalho Lana et al. 2021	2 Minute Walk Test (2MWT)	Step count/ mHealth devices (Google Fit, Health, STEPZ, Pacer, and Fitbit INC®)	**Validity (Pearson's correlation coefficient)** GoogleFit and STEZ were very highly correlated to 2MWT (*r* = 0.92, *r* = 0.91) Pacer and Fitbit INC® were highly correlated to 2MWT (*r* = 0.77, *r* = 0.82) Health was moderately correlated to 2MWT (*r* = 0.54)
Safarpour et al. 2022	MDS-revised Unified Parkinson's Disease Rating Scale (MDS-UPDRS)	Walking hours, gait speed and sway area/ wearable sensors Opals	**Validity (Pearson's correlation coefficient)** Statistically significant correlation between the in-person MDS-UPDRS PIGD subscore and the remote sensors values of gait speed, gait time combined with postural sway (*r* = 0.61)

## Discussion

The majority of studies identified for this review are feasibility or pilot studies (level V evidence), reflecting the novelty and preliminary exploration of the use of certain technologies and virtual care systems for remote assessment of PwN.

### Remote outcome measures

Studies reported on lab-based or expensive remote technology including haptic systems,^18,23^ Computer-Assisted Rehabilitation Environment system (CAREN),^55,56^ Actigraph,^40,62^ and immersive virtual reality.^28,41,42,44,48^ Results from this review also revealed that asynchronous technology (i.e., mHealth or wearable sensors using store-and-forward communication) was used more frequently than synchronous technology (i.e., videoconference) when assessing PwN. Importantly, this scoping review demonstrates that there is a significant gap in the determination of needed psychometric characteristics of remote assessment developed for people living with neurological conditions. Telerehabilitation is growing and is quicky being accepted as a way to reduce access barriers for people living with neurological conditions.^68–72^ The use of sensor technology including wearables, wireless motion sensors, and smart home technology is expanding, in fact, some authors have even identified remote assessment as its own field in telemedicine called “telesemiotic.”^73^ For instance, Cubo et al. used motion sensor to capture changes in motor symptoms for people living with Parkinson's^39^ while others have studied the use of home sensors to assess safety and night-time wandering with people living with dementia.^74^ Usability of a device is critical for self-monitoring, autonomous use, and self-efficacy.^75,76^ Emerging smart home technologies have the potential to improve independence, quality of life, and remote monitoring for people with disability.^77,78^ Access, cost, portability, and availability of teleplatforms must be considered as laboratory-grade devices or expensive remote assessments may pose a challenge for clinical adoption and implementation.

### Types of assessment as per the International Classification of Functioning, Disability and Health

Studies incorporating remote assessments of body impairments were mostly completed with people living with impairments from stroke, multiple sclerosis, and Parkinson's disease. Mobile applications and wearable sensors were most frequently used and were reported as easy to use. Specifically, people living with Parkinson's disease reported that mHealth applications were easy to use.^40^ The use of remote supervision to monitor changes in symptoms and inform prognosis is increasingly reported when monitoring symptoms of Parkinson's disease^36,39,46,47^ and could well be suited for other neurodegenerative conditions such as multiple sclerosis, amyotrophic lateral sclerosis, and muscular dystrophy.

Studies of activity and participation used wearable technologies to detect changes in activities such as mobility, gait, and upper extremity functional tasks. This supports findings from recent systematic reviews reporting on the use of mobile devices to assess gait in people with Parkinson's disease,^79^ to assess physical activity and mobility in PwN,^7^ and on the feasibility of using remote assessments for hand function in PwN.^80^ To date, a limited number of studies have targeted the use of remote assessment for social participation or community reintegration outcomes for PwN.^81,82^ Community integration and societal participation are crucial components of life satisfaction for PwN.^83^ Future research should, therefore, focus on the development of remote assessments targeting social participation for PwN. Defining the ICF construct being assessed in terms of body impairments, activity limitation, or participation restriction may facilitate the development and clinical use of remote assessment.

### Psychometric data

Only one-third (*n* = 17) of the studies documented the psychometric properties of the remote outcome measures used. Importantly, these psychometric properties were mostly established in a controlled and standardized environment such as a laboratory setting. Therefore, it is critical to determine similar properties in clinical and home-based environments. No studies reported the standard error of measurement, the data around floor and ceiling effects as well as measure responsiveness is lacking and only one study documented the MCID and clinical normative data, making it harder for clinicians to rely on remote assessments for treatment planning.

Moderate to strong correlations between in-person and remote assessment measures were reported in a small number of the studies. However, a lack of congruence between the assessments performed in-person and remotely was also frequently documented. For example, Tobler-Ammann et al.^23^ documented high test–retest reliability for the virtual version of the Nine Hole Peg Test (NHPT) but when they compared it to an in-person assessment with a similar construct such as the Box & Block Test (BBT), correlations were low for the number of transported cubes (*p* = −0.23) and dropped cubes (*p* = −0.12). It is unclear if this difference was observed due to a lack of correlation between the in-person BBT and the virtual NHPT or because one test was administered in-person and one was administered virtually. Similarly, Wang et al. reported that people living with Parkinson's disease reached less far when measured using a wearable sensor in a virtual environment compared to the in-person environment while completing the same task.^49^ Although the same measure could be administered in a virtual and in-person context, they may be measuring different constructs as described by the ICF. For example, the in-person BBT could be measuring an activity-related task, while the virtual reality version could be measuring body impairments such as the velocity of finger movements. Since psychometric properties may differ depending on the context within which the assessment is being administered, validity, reliability, diagnostic accuracy, and normative values associated with a clinical measure should be established independently for both in-person and remote assessments^84^ and within the context that it is being used.^85^ Identifying comparative psychometric norms between in-person and remote assessments is key to the successful and effective hybrid use of telerehabilitation. It is also critical that validated remote assessments specific to other neurological injuries not included in this review be made available.

### Limitations and opportunities

The terminology used as search terms, as well as by authors to describe remote assessment, is an important limitation to note as it might have limited the number of studies identified and may have excluded potentially eligible studies for this scoping review. For example, overarching terms for telehealth, mHealth and telemedicine were used as search terms in this review, which may have led to missing key studies which used specific terms or devices for telehealth assessments such as smartphone applications. Future studies should expand search terms to include specific technologies used in telehealth. Our initial search was completed in 2020 near the beginning of the COVID-19 pandemic and an updated search was performed in May 2022 (2 years into the pandemic). Due to the rapidly growing body of literature on this topic, future updates will be necessary.

## Conclusion

It is critical for healthcare providers to consider the neurological condition, the outcome being assessed as per the ICF, the type of remote monitoring, as well as teleplatform used when using remote assessment clinically. Results from our review demonstrated that the identification and reporting of psychometric data for remote clinical outcome measures is still novel and psychometric properties may differ when being assessed in clinical or home environments, and laboratory spaces. When possible, clinicians should use remote assessments which have been validated in a clinical or home space before adopting them within their clinical practice. It is necessary to establish the reliability and validity of remote assessments for each neurological condition to improve adoption and clinical implementation of assessment measures within various clinical contexts.

## Appendix 1

### Search strategy

#### CINAHL January 4, 2021, and May 9, 2022

1. TI (telerehabilitation/ or tele-rehabilitation/ or telemedicine/ or telehealth/ or eHealth/ or mHealth/ or teletherapy/ or teleconsultation/ or telemonitoring/ or telemetry/ or telecommunication/ or videoconferencing/ or telecare/ or videoconsultation/ or teleconcussion/ or telestroke/ or telepractice/ or telespeech/ or tele-OT/ or virtual rehabilitation/ or remote rehabilitation/ or distant therapy/ or distant physio/ or distant consultation/ or distant rehabilitation/ or remote consultation/ or virtual reality/) OR AB (telerehabilitation/ or tele-rehabilitation/ or telemedicine/ or telehealth/ or eHealth/ or mHealth/ or teletherapy/ or teleconsultation/ or telemonitoring/ or telemetry/ or telecommunication/ or videoconferencing/ or telecare/ or videoconsultation/ or teleconcussion/ or telestroke/ or telepractice/ or telespeech/ or tele-OT/ or virtual rehabilitation/ or remote rehabilitation/ or distant therapy/ or distant physio/ or distant consultation/ or distant rehabilitation/ or remote consultation/ or virtual reality/)
2. TI (neurologic/ or neurologic ambulation disorder/ or gait disorder/ or acquired brain injury/ or ABI/ or traumatic brain injury/ or brain injury/ or TBI/ or stroke/ or brain tumor/ or neurodegenerative/ or parkinson/ or multiple sclerosis/ or cerebral palsy/ or spinal cord injury/ or SCI/) OR AB (neurologic/ or neurologic ambulation disorder/ or gait disorder/ or acquired brain injury/ or ABI/ or traumatic brain injury/ or brain injury/ or TBI/ or stroke/ or brain tumor/ or neurodegenerative/ or parkinson/ or multiple sclerosis/ or cerebral palsy/ or spinal cord injury/ or SCI/)
3. TI ((rehabilitation/ or gait/ or ambulation/ or motor/ or function/ or walk/ or mobility/ or sit posture/ or stand posture/ or daily function/ or balance/ or strength/) N2 (evaluation/ or assessment/ or outcome measure/)) OR AB ((rehabilitation/ or gait/ or ambulation/ or motor/ or function/ or walk/ or mobility/ or sit posture/ or stand posture/ or daily function/ or balance/ or strength/) N2 (evaluation/ or assessment/ or outcome measure/))
4. #1 AND #2 AND #3

#### Medline (OVID) December 13, 2020, and May 9, 2022

1. (Telerehabilitation/ or telemedicine/ or telehealth or eHealth or mHealth or teletherapy or teleconsultation or telemonitoring or telemetry/ or telecommunication/ or videoconferencing/ or telecare or videoconsultation or teleconcussion or telestroke or telepractice or telespeech or tele-OT).ti,ab.
2. ((rehabilitation or gait or ambulation or motor or function or walk or mobility or sit posture or stand posture or daily function or balance or strength) adj5 (evaluation or assessment or outcome measure)).ti,ab.
3. ((distant adj2 care) or therapy or physio or consultation or rehabilitation or ((remote adj2 consultation) or rehabilitation) or (virtual adj2 rehabilitation) or virtual reality/ or ((tele adj2 rehabilitation) or OT or speech)).ti,ab.
4. (neurologic or neurologic ambulation disorder or gait disorder or acquired brain injury or ABI or traumatic brain injury or brain injury or TBI or stroke or brain tumor or neurodegenerative or parkinson or multiple sclerosis or cerebral palsy or spinal cord injury or SCI).ti,ab.
5. 1 and 2 and 3 and 4

#### PsychINFO December 13, 2020, and May 9, 2022

1. (Telerehabilitation/ or telemedicine/ or telehealth or eHealth or mHealth or teletherapy or teleconsultation or telemonitoring or telemetry/ or telecommunication/ or videoconferencing/ or telecare or videoconsultation or teleconcussion or telestroke or telepractice or telespeech or tele-OT).ti,ab.
2. ((rehabilitation or gait or ambulation or motor or function or walk or mobility or sit posture or stand posture or daily function or balance or strength) adj5 (evaluation or assessment or outcome measure)).ti,ab.
3. ((distant adj2 care) or therapy or physio or consultation or rehabilitation or ((remote adj2 consultation) or rehabilitation) or (virtual adj2 rehabilitation) or virtual reality/).mp. or ((tele adj2 rehabilitation) or OT or speech).ti,ab. [mp = title, abstract, heading word, table of contents, key concepts, original title, tests & measures, mesh]
4. (neurologic or neurologic ambulation disorder or gait disorder or acquired brain injury or ABI or traumatic brain injury or brain injury or TBI or stroke or brain tumor or neurodegenerative or parkinson or multiple sclerosis or cerebral palsy or spinal cord injury or SCI).ti,ab.
5. 1 and 2 and 3 and 4

#### EMBASE December 13, 2020, and May 9, 2022

1. (Telerehabilitation/ or telemedicine/ or telehealth or eHealth or mHealth or teletherapy or teleconsultation or telemonitoring or telemetry/ or telecommunication/ or videoconferencing/ or telecare or videoconsultation or teleconcussion or telestroke or telepractice or telespeech or tele-OT).ti,ab.
2. ((rehabilitation or gait or ambulation or motor or function or walk or mobility or sit posture or stand posture or daily function or balance or strength) adj5 (evaluation or assessment or outcome measure)).ti,ab.
3. ((distant adj2 care) or therapy or physio or consultation or rehabilitation or ((remote adj2 consultation) or rehabilitation) or (virtual adj2 rehabilitation) or virtual reality/ or ((tele adj2 rehabilitation) or OT or speech)).ti,ab.
4. (neurologic or neurologic ambulation disorder or gait disorder or acquired brain injury or ABI or traumatic brain injury or brain injury or TBI or stroke or brain tumor or neurodegenerative or parkinson or multiple sclerosis or cerebral palsy or spinal cord injury or SCI).ti,ab.
5. 1 and 2 and 3 and 4

#### Pubmed December 13, 2020, and May 9, 2022

1. (”tele-rehabilitation” OR “telerehabilitation” OR “telemedicine OR “telehealth"OR “eHealth” OR “mHealth” OR “teletherapy” OR “teleconsultation” OR “telemonitoring” OR “telemetry” OR “tele homecare” OR “telecommunication” OR “videoconferencing” OR “telecare” OR “videoconsultation” OR “teleconcussion” OR “telestroke” OR “telepractice” OR “telespeech” OR “virtual rehabilitation” OR “remote rehabilitation”) [Title/Abstract] OR (“distant"[All Fields] AND “therapy"[Title/Abstract]) OR (“distant"[All Fields] AND “physio"[Title/Abstract]) OR “distant consultation"[Title/Abstract] OR “distant rehabilitation"[Title/Abstract] OR “remote consultation"[Title/Abstract] OR “virtual reality"[Title/Abstract])
2. (”neurologic” [Title/Abstract] OR ((“neurologic manifestations"[MeSH Terms] OR (“neurologic"[All Fields] AND “manifestations"[All Fields]) OR “neurologic manifestations"[All Fields] OR “neurologic"[All Fields] OR “neurological"[All Fields] OR “neurologically"[All Fields]) AND “ambulation disorder"[Title/Abstract]) OR (“gait disorder” OR “acquired brain injury” OR “ABI” OR “traumatic brain injury” OR “brain injury"OR “TBI” OR “stroke” OR “brain tumor” OR “neurodegenerative” OR “parkinson” OR “multiple sclerosis” OR “cerebral palsy” OR “spinal cord injury” OR “SCI”) [Title/Abstract]
3. (”rehabilitation evaluation” OR “rehabilitation assessment” OR “rehabilitation outcome measure” OR “gait evaluation” OR “gait assessment” OR ((“gait"[MeSH Terms] OR “gait"[All Fields]) [Title/Abstract] AND “outcome measure"[Title/Abstract]) OR ((“ambulant"[All Fields] OR “ambulate"[All Fields] OR “ambulated"[All Fields] OR “ambulates"[All Fields] OR “ambulating"[All Fields] OR “ambulations"[All Fields] OR “ambulator"[All Fields] OR “ambulators"[All Fields] OR “walking"[MeSH Terms] OR “walking"[All Fields] OR “ambulation"[All Fields]) AND “evaluation"[Title/Abstract]) OR ((“ambulant"[All Fields] OR “ambulate"[All Fields] OR “ambulated"[All Fields] OR “ambulates"[All Fields] OR “ambulating"[All Fields] OR “ambulations"[All Fields] OR “ambulator"[All Fields] OR “ambulators"[All Fields] OR “walking"[MeSH Terms] OR “walking"[All Fields] OR “ambulation"[All Fields]) AND “assessment"[Title/Abstract]) OR ((“ambulant"[All Fields] OR “ambulate"[All Fields] OR “ambulated"[All Fields] OR “ambulates"[All Fields] OR “ambulating"[All Fields] OR “ambulations"[All Fields] OR “ambulator"[All Fields] OR “ambulators"[All Fields] OR “walking"[MeSH Terms] OR “walking"[All Fields] OR “ambulation"[All Fields]) AND “outcome measure"[Title/Abstract]) OR ((“walking"[MeSH Terms] OR “walking"[All Fields] OR “walk"[All Fields]) AND “evaluation"[Title/Abstract]) OR “walk assessment"[Title/Abstract] OR ((“walking"[MeSH Terms] OR “walking"[All Fields] OR “walk"[All Fields]) AND “outcome measure"[Title/Abstract]) OR “motor evaluation"[Title/Abstract] OR “motor assessment"[Title/Abstract] OR “motor outcome measure"[Title/Abstract] OR “function assessment"[Title/Abstract] OR “function evaluation"[Title/Abstract] OR “function outcome measure"[Title/Abstract] OR “mobility evaluation"[Title/Abstract] OR “mobility assessment"[Title/Abstract] OR “mobility outcome measure"[Title/Abstract] OR ((“sitting position"[MeSH Terms] OR (“sitting"[All Fields] AND “position"[All Fields]) OR “sitting position"[All Fields] OR “sit"[All Fields]) AND “posture evaluation"[Title/Abstract]) OR ((“sitting position"[MeSH Terms] OR (“sitting"[All Fields] AND “position"[All Fields]) OR “sitting position"[All Fields] OR “sit"[All Fields]) AND “posture assessment"[Title/Abstract]) OR (((“sitting position"[MeSH Terms] OR (“sitting"[All Fields] AND “position"[All Fields]) OR “sitting position"[All Fields] OR “sit"[All Fields]) AND (“postural"[All Fields] OR “posturally"[All Fields] OR “posture"[MeSH Terms] OR “posture"[All Fields] OR “postures"[All Fields] OR “postured"[All Fields] OR “posturing"[All Fields])) AND “outcome measure"[Title/Abstract]) OR ((“stand"[All Fields] OR “standing position"[MeSH Terms] OR (“standing"[All Fields] AND “position"[All Fields]) OR “standing position"[All Fields] OR “standing"[All Fields] OR “standings"[All Fields] OR “stands"[All Fields]) AND “posture evaluation"[Title/Abstract]) OR ((“stand"[All Fields] OR “standing position"[MeSH Terms] OR (“standing"[All Fields] AND “position"[All Fields]) OR “standing position"[All Fields] OR “standing"[All Fields] OR “standings"[All Fields] OR “stands"[All Fields]) AND “posture assessment"[Title/Abstract]) OR (((“stand"[All Fields] OR “standing position"[MeSH Terms] OR (“standing"[All Fields] AND “position"[All Fields]) OR “standing position"[All Fields] OR “standing"[All Fields] OR “standings"[All Fields] OR “stands"[All Fields]) AND (“postural"[All Fields] OR “posturally"[All Fields] OR “posture"[MeSH Terms] OR “posture"[All Fields] OR “postures"[All Fields] OR “postured"[All Fields] OR “posturing"[All Fields])) AND “outcome measure"[Title/Abstract]) OR ((“dailies"[All Fields] OR “daily"[All Fields]) AND “function evaluation"[Title/Abstract]) OR ((“dailies"[All Fields] OR “daily"[All Fields]) AND “function assessment"[Title/Abstract]) OR ((“dailies"[All Fields] OR “daily"[All Fields]) AND “function outcome measure"[Title/Abstract]) OR “balance evaluation"[Title/Abstract] OR “balance assessment"[Title/Abstract] OR “balance outcome measure"[Title/Abstract] OR “strength evaluation"[Title/Abstract] OR “strength assessment"[Title/Abstract] OR ((“strength"[All Fields] OR “strengths"[All Fields]) AND “outcome measure"[Title/Abstract]))
4. #1 AND #2 AND #3

#### Cochrane December 15, 2020, and May 9, 2022

(neurologic or neurologic ambulation disorder or gait disorder or acquired brain injury or ABI or traumatic brain injury or brain injury or TBI or stroke or brain tumor or neurodegenerative or parkinson or multiple sclerosis or cerebral palsy or spinal cord injury or SCI):ti,ab,kw AND (rehabilitation or gait or ambulation or walk or motor or function or mobility or sit posture or stand posture or daily function or balance or strength) near (evaluation or assessment or outcome measure):ti,ab,kw AND (telerehabilitation or telerehabilitation or telemedicine or telehealth or eHealth or mHealth or teletherapy or teleconsultation or telemonitoring or telemetry or tele homecare or telecommunication or videoconferencing or telecare or videoconsultation or teleconcussion or telestroke or telepractice or telespeech or tele-OT or virtual rehabilitation or remote rehabilitation or distant therapy or distant physio or distant consultation or distant rehabilitation or remote consultation or virtual reality):ti,ab,kw (Word variations have been searched)

## References

[1] World Health Organization (WHO). Recommendations on digital interventions for health system strengthening. WHO 2019; 2: 1–3. 31162915

[2] Parmanto B Saptono A . Telerehabilitation: state-of-the-art from an informatics perspective. Int J Telerehabil 2009; 1: 73–84. 25945164 10.5195/ijt.2009.6015 PMC4296781

[3] Bhaskar S Bradley S Chattu VK , et al. Telemedicine as the new outpatient clinic gone digital: Position Paper From the Pandemic Health System REsilience PROGRAM (REPROGRAM) International Consortium (Part 2). Front Public Health 2020; 8 (article 410). 10.3389/fpubh.2020.00410 PMC7505101 33014958

[4] Chirra M Marsili L Wattley L , et al. Telemedicine in neurological disorders: opportunities and challenges. Telemed J E Health 2019; 25: 541–550. 30136898 10.1089/tmj.2018.0101 PMC6664824

[5] Mani S Sharma S Omar B , et al. Validity and reliability of internet-based physiotherapy assessment for musculoskeletal disorders: a systematic review. J Telemed Telecare 2017; 23: 379–391. 27036879 10.1177/1357633X16642369

[6] Peyrusqué E Granet J Pageaux B , et al. Assessing physical performance in older adults during isolation or lockdown periods: web-based video conferencing as a solution. J Nutr Health Aging 2022; 26: 52–56. 35067703 10.1007/s12603-021-1699-y PMC8590923

[7] Block VAJ Pitsch E Tahir P , et al. Remote physical activity monitoring in neurological disease: a systematic review. PLoS One 2016; 11: 1–41. 10.1371/journal.pone.0154335 PMC4849800 27124611

[8] Madhavan S Sivaramakrishnan A Bowden MG , et al. Commentary: remote assessments of gait and balance—implications for research during and beyond COVID-19. Top Stroke Rehabil 2022; 29: 74–81. 33596774 10.1080/10749357.2021.1886641 PMC8371083

[9] Jansen-Kosterink S Dekker-van Weering M van Velsen L . Patient acceptance of a telemedicine service for rehabilitation care: a focus group study. Int J Med Inform 2019; 125: 22–29. 30914177 10.1016/j.ijmedinf.2019.01.011

[10] Wentink M Van Bodegom-Vos L Brouns B , et al. How to improve eRehabilitation programs in stroke care? A focus group study to identify requirements of end-users. BMC Med Inform Decis Mak 2019; 19: 1–11. 31349824 10.1186/s12911-019-0871-3 PMC6660703

[11] Peters S Botero M Evers A , et al. Development and feasibility of a modified Fugl-Meyer lower extremity assessment for telerehabilitation: a pilot study. Pilot Feasibility Stud 2021; 7: 1–9. 34099053 10.1186/s40814-021-00862-8 PMC8182356

[12] Moccia M Lanzillo R Brescia Morra V , et al. Assessing disability and relapses in multiple sclerosis on tele-neurology. Neurol Sci 2020; 41: 1369–1371. 32440979 10.1007/s10072-020-04470-x PMC7241064

[13] O’Neil J Barnes K Donnelly EM , et al. Identification and description of balance, mobility, and gait assessments conducted via telerehabilitation for individuals with neurological conditions: protocol for a scoping review. JMIR Res Protoc 2021; 10: 1–7. 10.2196/27186 PMC8704120 34889765

[14] Arksey and O’Malley. Scoping studies: towards a methodological framework. Int J Soc Res Methodol 2005; 8: 19–32.

[15] Colquhoun HL Levac D O’Brien KK , et al. Scoping reviews: time for clarity in definition, methods, and reporting. J Clin Epidemiol 2014; 67: 1291–1294. 25034198 10.1016/j.jclinepi.2014.03.013

[16] Butler C Darrah J Adams R , et al. Effects of neurodevelopmental treatment (NDT) for cerebral palsy: an AACPDM evidence report. Dev Med Child Neurol 2001; 43: 778–790. 11730153 10.1017/s0012162201001414

[17] McHugh ML . Lessons in biostatistics interrater reliability : the kappa statistic. Biochem Med 2012; 22: 276–282. PMC3900052 23092060

[18] Broeren J Rydmark M Björkdahl A , et al. Assessment and training in a 3-dimensional virtual environment with haptics: a report on 5 cases of motor rehabilitation in the chronic stage after stroke. Neurorehabil Neural Repair 2007; 21: 180–189. 17312093 10.1177/1545968306290774

[19] Trinh T Scheuer SE Thompson-Butel AG , et al. Cardiovascular fitness is improved poststroke with upper-limb Wii-based movement therapy but not dose-matched constraint therapy. Top Stroke Rehabil 2016; 23: 208–216. 26907502 10.1080/10749357.2016.1138672

[20] Thielbar KO Lord TJ Fischer HC , et al. Training finger individuation with a mechatronic-virtual reality system leads to improved fine motor control post-stroke. J Neuroeng Rehabil 2014; 11: 1–11. 25542201 10.1186/1743-0003-11-171 PMC4292811

[21] Sungmin C Won-Seok K Nam-jong P , et al. Upper-limb function assessment using VBBTs for stroke patients. IEEE Comput Soc 2016; 36: 70–78. 10.1109/MCG.2015.2 25585413

[22] Luque-Moreno C Oliva-Pascual-Vaca A Kiper P , et al. Virtual reality to assess and treat lower extremity disorders in post-stroke patients. Methods Inf Med 2016; 55: 89–92. 26660161 10.3414/ME14-02-0020

[23] Tobler-Ammann BC De Bruin ED Fluet MC , et al. Concurrent validity and test-retest reliability of the virtual peg insertion test to quantify upper limb function in patients with chronic stroke. J Neuroeng Rehabil 2016; 13: 1–14. 26801395 10.1186/s12984-016-0116-y PMC4724098

[24] Wittmann F Held JP Lambercy O , et al. Self-directed arm therapy at home after stroke with a sensor-based virtual reality training system. J Neuroeng Rehabil 2016; 13: 1–10. 27515583 10.1186/s12984-016-0182-1 PMC4982313

[25] Perez-Marcos D Chevalley O Schmidlin T , et al. Increasing upper limb training intensity in chronic stroke using embodied virtual reality: a pilot study. J Neuroeng Rehabil 2017; 14: 1–14. 29149855 10.1186/s12984-017-0328-9 PMC5693522

[26] Wall KJ Cumming TB Koenig ST , et al. Using technology to overcome the language barrier: the cognitive assessment for aphasia app. Disabil Rehabil 2018; 40: 1333–1344. 28271907 10.1080/09638288.2017.1294210

[27] Hung JW Chou CX Chang YJ , et al. Comparison of Kinect2Scratch game-based training and therapist-based training for the improvement of upper extremity functions of patients with chronic stroke: a randomized controlled single-blinded trial. Eur J Phys Rehabil Med 2019; 55: 542–550. 30781936 10.23736/S1973-9087.19.05598-9

[28] Thielbar KO Triandafilou KM Barry AJ , et al. Home-based upper extremity stroke therapy using a multiuser virtual reality environment: a randomized trial. Arch Phys Med Rehabil 2020; 101: 196–203. 31715140 10.1016/j.apmr.2019.10.182

[29] de Rooij IJM van de Port IGL Punt M , et al. Effect of virtual reality gait training on participation in survivors of subacute stroke: a randomized controlled trial. Phys Ther 2021; 101: 1–10. 10.1093/ptj/pzab051 PMC8122468 33594443

[30] Szturm T Imran Z Pooyania S , et al. Evaluation of a game based telerehabilitation platform for in-home therapy of hand-arm function post stroke: feasibility study. PM R 2021; 13: 45–54. 32107868 10.1002/pmrj.12354

[31] Shazowee Edgerton S . A pilot study investigating employee utilization of corporate telehealth services. Perspect Health Inf Manag 2017; 14: 1–14. PMC5653955 29118684

[32] Combs SA Finley MA Henss M , et al. Effects of a repetitive gaming intervention on upper extremity impairments and function in persons with chronic stroke: a preliminary study. Disabil Rehabil 2012; 34: 1291–1298. 22324473 10.3109/09638288.2011.641660

[33] Jack D Boian R Member S , et al. Virtual reality-enhanced stroke rehabilitation. IEEE Trans Neural Syst Rehabil Eng 2001; 9: 308–318. 11561668 10.1109/7333.948460

[34] Fischer HC Stubblefield K Kline T , et al. Hand rehabilitation following stroke: a pilot study of assisted finger extension training in a virtual environment. Top Stroke Rehabil 2007; 14: 1–12. 10.1310/tsr1401-1 17311785

[35] Giansanti D Macellari V Giovanni M . Telemonitoring and telerehabilitation of patients with Parkinson’s disease: health technology assessment of a novel wearable step counter. Telemed J E Health 2008; 14: 76–83. 10.1089/tmj.2007.0019 18328028

[36] Westin J Schiavella M Memedi M , et al. Validation of a home environment test battery for supporting assessments in advanced Parkinson’s disease. Neurol Sci 2012; 33: 831–838. 22068219 10.1007/s10072-011-0844-5

[37] Gilat M Shine JM Bolitho SJ , et al. Variability of stepping during a virtual reality paradigm in Parkinson’s disease patients with and without freezing of gait. PLoS One 2013; 8: 1–6. 10.1371/journal.pone.0066718 PMC3689740 23805270

[38] Arora S Venkataraman V Zhan A , et al. Detecting and monitoring the symptoms of Parkinson’s disease using smartphones: a pilot study. Parkinsonism Relat Disord 2015; 21: 650–653. 25819808 10.1016/j.parkreldis.2015.02.026

[39] Cubo E Mariscal N Solano B , et al. Prospective study on cost-effectiveness of home-based motor assessment in Parkinson’s disease. J Telemed Telecare 2017; 23: 328–338. 27000142 10.1177/1357633X16638971

[40] Horin AP McNeely ME Harrison EC , et al. Usability of a daily mHealth application designed to address mobility, speech and dexterity in Parkinson’s disease. Neurodegener Dis Manag 2019; 9: 97–105. 30998100 10.2217/nmt-2018-0036 PMC7026767

[41] Lheureux A Lebleu J Frisque C , et al. Immersive virtual reality to restore natural long-range autocorrelations in Parkinson’s disease patients’ gait during treadmill walking. Front Physiol 2020; 11: 1–9. 33071825 10.3389/fphys.2020.572063 PMC7538859

[42] Oña ED Jardón A Cuesta-Gómez A , et al. Validity of a fully-immersive VR-based version of the box and blocks test for upper limb function assessment in Parkinson’s disease. Sensors (Switzerland) 2020; 20: 1–17. 10.3390/s20102773 PMC7285781 32414177

[43] Singh S Xu W . Robust detection of Parkinson’s disease using harvested smartphone voice data: a telemedicine approach. Telemed J E Health 2020; 26: 327–334. 31033397 10.1089/tmj.2018.0271 PMC7071066

[44] de Carvalho Lana R Ribeiro de Paula A Souza Silva AF , et al. Validity of mHealth devices for counting steps in individuals with Parkinson's disease. J Bodyw Mov Ther. 2021;28:496–501. 34776185 10.1016/j.jbmt.2021.06.018

[45] Hawkins KE Paul SS Chiarovano E , et al. Using virtual reality to assess vestibulo-visual interaction in people with Parkinson’s disease compared to healthy controls. Exp Brain Res 2021; 239: 3553–3564. 34562106 10.1007/s00221-021-06219-0

[46] Borzì L Mazzetta I Zampogna A , et al. Single Inertial Sensor. 2022;1–22. 10.3390/s22020412 PMC8778464 35062375

[47] Safarpour D Dale ML Shah VV , et al. Surrogates for rigidity and PIGD MDS-UPDRS subscores using wearable sensors. Gait Posture 2022; 91: 186–191. 34736096 10.1016/j.gaitpost.2021.10.029 PMC8671321

[48] Arias P Robles-García V Sanmartín G , et al. Virtual reality as a tool for evaluation of repetitive rhythmic movements in the elderly and Parkinson’s disease patients. PLoS One 2012; 7: e30021. 10.1371/journal.pone.0030021 PMC3261172 22279559

[49] Wang CY Hwang WJ Fang JJ , et al. Comparison of virtual reality versus physical reality on movement characteristics of persons with Parkinson’s disease: effects of moving targets. Arch Phys Med Rehabil 2011; 92: 1238–1245. 21718966 10.1016/j.apmr.2011.03.014

[50] Westin J Dougherty M Nyholm D , et al. A home environment test battery for status assessment in patients with advanced Parkinson’s disease. Comput Methods Programs Biomed 2010; 98: 27–35. 19740563 10.1016/j.cmpb.2009.08.001

[51] Killane I Fearon C Newman L , et al. Dual motor-cognitive virtual reality training impacts dual-task performance in freezing of gait. IEEE J Biomed Health Inform 2015; 19: 1855–1861. 26394439 10.1109/JBHI.2015.2479625

[52] Vargas BB Shepard M Hentz JG , et al. Feasibility and accuracy of teleconcussion for acute evaluation of suspected concussion. Neurology 2017; 88: 1580–1583. 28341642 10.1212/WNL.0000000000003841

[53] Zhang L Abreu BC Masel B , et al. Virtual reality in the assessment of selected cognitive function after brain injury. Am J Phys Med Rehabil 2001; 80: 597–604. 11475481 10.1097/00002060-200108000-00010

[54] Theodoros D Russell TG Hill A , et al. Assessment of motor speech disorders online: a pilot study. J Telemed Telecare 2003; 9: 66–68. 10.1258/135763303322596318 14728766

[55] Ràbago CA Wilken JM . Application of a mild traumatic brain injury rehabilitation program in a virtual realty environment: a case study. J Neurol Phys Ther 2011; 35: 185–193. 22027473 10.1097/NPT.0b013e318235d7e6

[56] Sessoms PH Gottshall KR Sturdy J , et al. Head stabilization measurements as a potential evaluation tool for comparison of persons with TBI and vestibular dysfunction with healthy controls. Mil Med 2015; 180: 3S. 10.7205/MILMED-D-14-00386 25747644

[57] Wright WG Tierney RT McDevitt J . Visual-vestibular processing deficits in mild traumatic brain injury. J Vestib Res 2017; 27: 27–37. 28387693 10.3233/VES-170607

[58] Salisbury JP Keshav NU Sossong AD , et al. Concussion assessment with smartglasses: validation study of balance measurement toward a lightweight, multimodal, field-ready platform. JMIR Mhealth Uhealth 2018; 6: 1–14. 10.2196/mhealth.8478 PMC5801523 29362210

[59] Cuthbert JP . Virtual Reality-Based Therapy for the Treatment of Balance Deficits in Patients Receiving Inpatient Rehabilitation for Traumatic Brain Injury. 2012. 10.3109/02699052.2013.860475 24456057

[60] Leocani L Comi E Annovazzi P , et al. Impaired short-term motor learning in multiple sclerosis: evidence from virtual reality. Am Soc Neurorehabil 2007; 21: 273–278. 10.1177/1545968306294913 17351084

[61] Kane RL Bever CT Ehrmantraut M , et al. Teleneurology in patients with multiple sclerosis: EDSS ratings derived remotely and from hands-on examination. J Telemed Telecare 2008; 14: 190–194. 18534953 10.1258/jtt.2008.070904

[62] Sola-Valls N Blanco Y Sepúlveda M , et al. Walking function in clinical monitoring of multiple sclerosis by telemedicine. J Neurol 2015; 262: 1706–1713. 25957639 10.1007/s00415-015-7764-x

[63] Maillart E Labauge P Cohen M , et al. MSCopilot, a new multiple sclerosis self-assessment digital solution: results of a comparative study versus standard tests. Eur J Neurol 2020; 27: 429–436. 31538396 10.1111/ene.14091

[64] Finkelstein J Liu J . Designing telerehabilitation system for multipronged exercise in patients with multiple sclerosis. Stud Health Technol Inform 2018; 254: 16–23. 30306953

[65] Kowalczewski J Chong SL Galea M , et al. In-home tele-rehabilitation improves tetraplegic hand function. Neurorehabil Neural Repair 2011; 25: 412–422. 21372246 10.1177/1545968310394869

[66] Trincado-Alonso F Dimbwadyo-Terrer I De Los Reyes-Guzmán A , et al. Kinematic metrics based on the virtual reality system Toyra as an assessment of the upper limb rehabilitation in people with spinal cord injury. Biomed Res Int 2014: 904985. Epub. 10.1155/2014/904985 PMC4017839 24895627

[67] An Y Park C . The effects of virtual soccer game on balance, gait function, and kick speed in chronic incomplete spinal cord injury: a randomized controlled trial. Spinal Cord 2022; 60: 504–509. 10.1038/s41393-021-00745-y 34999726

[68] Caughlin S Mehta S Corriveau H , et al. Implementing telerehabilitation after stroke: lessons learned from Canadian trials. Telemed J E Health 2020; 26: 710–719. 31633454 10.1089/tmj.2019.0097

[69] Kairy D Veras M Archambault P , et al. Maximizing post-stroke upper limb rehabilitation using a novel telerehabilitation interactive virtual reality system in the patient’s home. Contemp Clin Trials 2015; 47: 49–53. 26655433 10.1016/j.cct.2015.12.006

[70] O’Neil J Pelletier L Bilodeau M , et al. A physiotherapist’s perception of their own behavior compared to the perception of their behavior by persons with TBI within the context of telerehabilitation: a self-determination theory perspective. Physiother Theory Pract 2022: 1–12. Online. 10.1080/09593985.2022.2046219 35220861

[71] Touchett H Apodaca C Siddiqui S , et al. Current approaches in telehealth and telerehabilitation for spinal cord injury (TeleSCI). Curr Phys Med Rehabil Rep 2022; 10: 77–88. 35493027 10.1007/s40141-022-00348-5 PMC9039273

[72] Sechrist S Lavoie S Khong CM , et al. Telemedicine using an iPad in the spinal cord injury population: a utility and patient satisfaction study. Spinal Cord Ser Cases 2018; 4: 71. 10.1038/s41394-018-0105-4 PMC6082908 30131874

[73] Brizio A Faure V Baudino F . Medical semiotics in the telemedicine era: The birth of telesemiotics. Int J Med Inform 2022; 157: 104573. 10.1016/j.ijmedinf.2021.104573 34753040

[74] Ault L Goubran R Wallace B , et al. Smart home technology solution for night-time wandering in persons with dementia. J Rehabil Assist Technol Eng 2020; 7: 205566832093859. 10.1177/2055668320938591 PMC8855416 35186320

[75] Albani G Ferraris C Nerino R , et al. An integrated multi-sensor approach for the remote monitoring of Parkinson’s disease. Sensors (Switzerland) 2019; 19: 1–18. 10.3390/s19214764 PMC6864792 31684020

[76] Sawesi S Rashrash M Phalakornkule K , et al. The impact of information technology on patient engagement and health behavior change: a systematic review of the literature. JMIR Med Inform 2016; 4: e1. 26795082 10.2196/medinform.4514 PMC4742621

[77] Jamwal R Jarman HK Roseingrave E , et al. Smart home and communication technology for people with disability: a scoping review. Disabil Rehabil Assist Technol 2022; 17: 624–644. 10.1080/17483107.2020.1818138 32924660

[78] Talal M Zaidan AA Zaidan BB , et al. Smart home-based IoT for real-time and secure remote health monitoring of triage and priority system using body sensors: multi-driven systematic review. J Med Syst 2019; 43: 42. 10.1007/s10916-019-1158-z 30648217

[79] Abou L Peters J Wong E , et al. Gait and balance assessments using smartphone applications in Parkinson’s disease: a systematic review. J Med Syst 2021; 45: 1–20. 10.1007/s10916-021-01760-5 PMC8364438 34392429

[80] Gopal A Hsu WY Allen DD , et al. Remote assessments of hand function in neurological disorders: systematic review. JMIR Rehabil Assist Technol 2022; 9: e33157. 10.2196/33157 PMC8943610 35262502

[81] Pugh MJ Swan AA Carlson KF , et al. Traumatic brain injury severity, comorbidity, social support, family functioning, and community reintegration among veterans of the Afghanistan and Iraq wars. Arch Phys Med Rehabil 2018; 99: S40–S49. 28648681 10.1016/j.apmr.2017.05.021

[82] Maas JJL De Vries NM Bloem BR , et al. Design of the PERSPECTIVE study: PERsonalized SPEeCh therapy for actIVE conversation in Parkinson’s disease (randomized controlled trial). Trials 2022; 23: 1–11. 35395953 10.1186/s13063-022-06160-9 PMC8990485

[83] Teasell R Salbach NM Foley N , et al. Canadian Stroke best practice recommendations: rehabilitation, recovery, and community participation following stroke. Part one: rehabilitation and recovery following stroke; 6th edition update 2019. Int J Stroke 2020: 763–788. 31983296 10.1177/1747493019897843

[84] Russell TG Martin-Khan M Khan A , et al. Method-comparison studies in telehealth: study design and analysis considerations. J Telemed Telecare 2017; 23: 797–802. 28893117 10.1177/1357633X17727772

[85] Montes J Eichinger KJ Pasternak A , et al. A post pandemic roadmap toward remote assessment for neuromuscular disorders: limitations and opportunities. Orphanet J Rare Dis 2022; 17: 1–7. 34983609 10.1186/s13023-021-02165-w PMC8726521

